# Downregulation of miR-200c stabilizes XIAP mRNA and contributes to invasion and lung metastasis of bladder cancer

**DOI:** 10.1080/19336918.2019.1633851

**Published:** 2019-06-26

**Authors:** Honglei Jin, Lei Xue, Lan Mo, Dongyun Zhang, Xirui Guo, Jiheng Xu, Jingxia Li, Minggang Peng, Xuewei Zhao, Minghao Zhong, Dazhong Xu, Xue-Ru Wu, Haishan Huang, Chuanshu Huang

**Affiliations:** a Nelson Institute of Environmental Medicine and Department of Environmental Medicine, New York University School of Medicine, Tuxedo, NY, USA; b Department of Thoracic Surgery, Changzheng Hospital, Second Military Medical University, Shanghai, China; c Department of Pathology, New York Medical College, Valhalla, NY, USA; d Departments of Urology and Pathology, New York University School of Medicine, New York, NY, USA; e Department of Environmental Medicine, VA Medical Center in Manhattan, New York University, New York, NY, USA; f Zhejiang Provincial Key Laboratory for Technology & Application of Model Organisms, School of Life Sciences, Wenzhou Medical University, Wenzhou, Zhejiang, China

**Keywords:** XIAP, mir-200c, bladder cancer, invasion/metastasis, CREB inactivation

## Abstract

Our previous studies have demonstrated that XIAP promotes bladder cancer metastasis through upregulating RhoGDIβ/MMP-2 pathway. However, the molecular mechanisms leading to the XIAP upregulation was unclear. In current studies, we found that XIAP was overexpressed in human high grade BCs, high metastatic human BCs, and in mouse invasive BCs. Mechanistic studies indicated that XIAP overexpression in the highly metastatic T24T cells was due to increased mRNA stability of XIAP that was mediated by downregulated miR-200c. Moreover, the downregulated miR-200c was due to CREB inactivation, while miR-200c downregulation reduced its binding to the 3’-UTR region of XIAP mRNA. Collectively, our results demonstrate the molecular basis leading to XIAP overexpression and its crucial role in BC invasion.

## Introduction

Bladder cancer (BC) is a malignancy of the urinary tract, and is the fourth and eighth most commonly diagnosed cancer in males and females, respectively, in the USA [1,2]. Unlike non-lethal superficial tumors, invasive/metastatic malignant tumors contribute to nearly 100% of BC-related deaths [3]. Given the public health need produced by BC invasion and metastasis, there is urgency in understanding and counteracting the molecular basis for BC invasion and metastasis.

X-linked inhibitor of apoptosis protein (XIAP) is a member of the inhibitor of apoptosis proteins (IAP) family, and is the main suppressor of apoptosis [4,5]. In addition to numerous studies elucidating the mechanisms of anti-apoptotic function of XIAP, our recent studies have revealed several non-apoptosis-related functions of XIAP, such as upregulation of Cyclin D1 to promote BC cell growth [6] and colon cancer cell invasion *via* inhibition of RhoGDIα SUMOylation at lys-138 [7]. XIAP functions as a metastatic driver by activation of the NFκB pathway *via*itsE3 ligase activity in human prostate cancer cells [8]. Furthermore, XIAP can promote bladder epithelial cell malignant transformation through inhibition of p63α translation [9]. Our newly research reported that XIAP promotes bladder cancer metastasis through upregulating RhoGDIβ/MMP-2 pathway [10,11]. But the molecular mechanisms leading to this XIAP alteration during BC metastasis have not been explored.

MiR-200c is a member of the miR-200 family that has five members divided into two clusters: one containing MiR-200a, miR-200b, and miR-429, while the other cluster contains miR-200c and miR-141 [12]. All miR-200 family members have been reported to be associated with regulating cancer metastasis [12], and act as considerable modulators in the process of epithelial-to-mesenchymal transition (EMT), which is a cell development regulatory process that promotes tumor development and metastasis [13,14]. Furthermore, previous reports have demonstrated that miR-200c could suppress metastasis, *via* mechanisms such as inhibition of MMP-3 expression to abolish ovarian cancer metastasis [15] and downregulation of E-cadherin to affect EMT in human renal cell carcinoma [16]. MiR-200c is also reported to target the 3ʹUTR region of XIAP resulting in proliferation inhibition and promotion of apoptosis of triple-negative breast cancer cells [17].

In the current study, we found that XIAP expression was positively correlated to tumor grade and muscle-invasive tumors, and we also elucidated the molecular mechanisms of ectopic expression of XIAP in BC invasion and metastasis. Specifically, we demonstrated that XIAP was highly expressed in highly invasive BCs through elevated mRNA stability. We also identified that miR-200c could directly target the 3ʹ-UTR region of xiap mRNA, reducing the stability of xiap mRNA. Moreover, Inactivation of CREB, as the transcription factor of miR-200c, resulted in the downregulation of miR-200c transcription, and led to XIAP overexpression and promotion of BC invasion and metastasis.

## Results

### XIAP expression is positively correlated with bladder cancer metastasis

**Figure 1.**
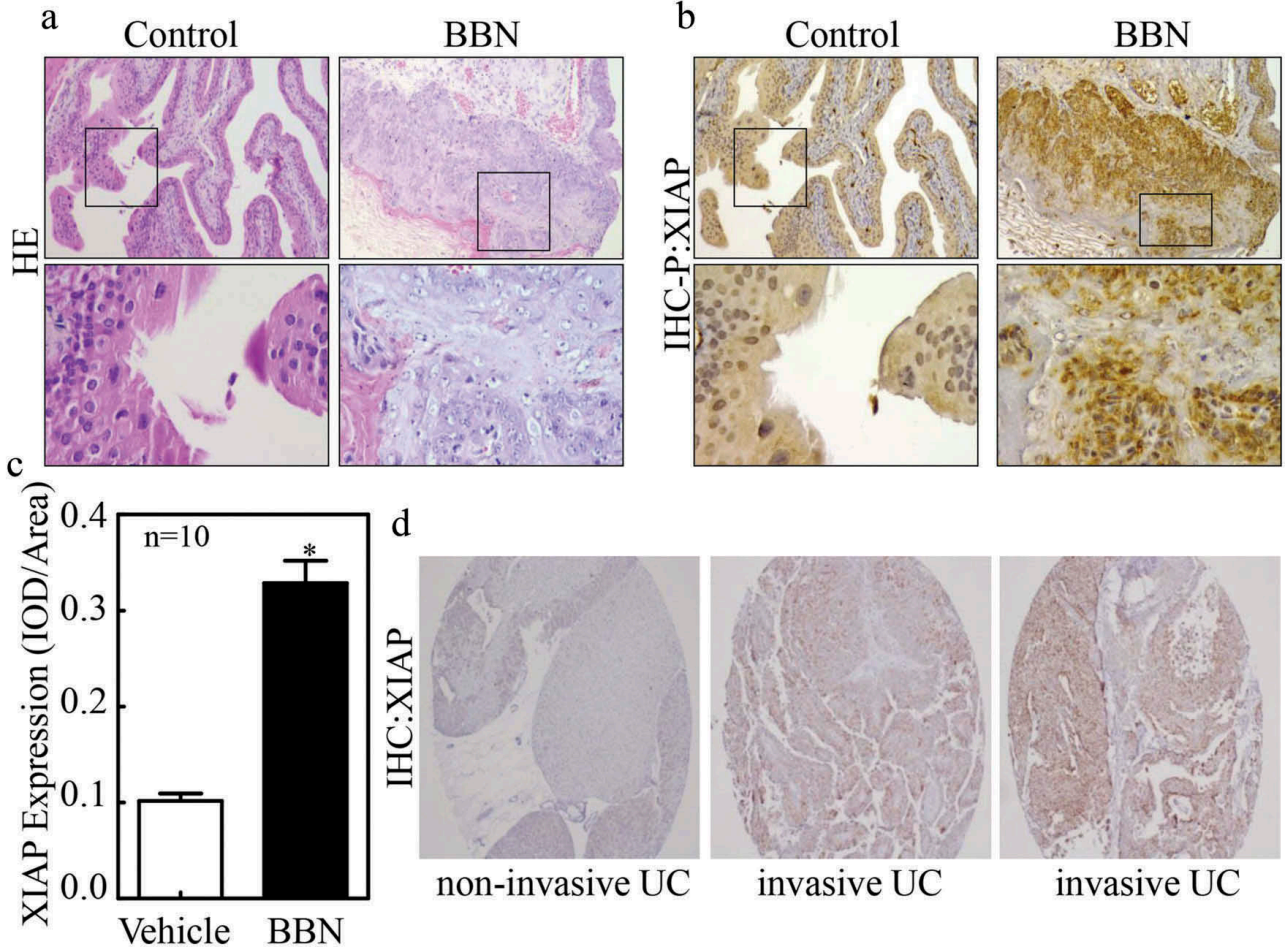
XIAP expression is positively correlated with bladder cancer invasion. (a) After 180 days treatment with or without BBN, the bladder tissues from indicated mice were analyzed by H&E staining; (b) IHC was also employed to evaluate XIAP expression in bladder tissues from indicated mice; and (c) the optical density was calculated as described in ‘Materials and Methods’. The results are presented as mean ± SD from at least triplicate experiments and an asterisk (*) indicates a significant difference (*P* < 0.05). (d) Human urothelial carcinoma tissue array was employed to evaluate XIAP expression.

XIAP is well known as an anti-apoptosis protein; however, it has other functions unrelated to apoptosis [6–9]. Our recent study demonstrated that XIAP promotes BC metastasis, but the molecular mechanism of XIAP upregulation in BC remains unclear. To explore this, we performed immunohistochemistry (IHC) staining to examine the expression of XIAP in N-butyl-N-(4-hydroxybutyl) nitrosamine (BBN)-induced mouse invasive bladder cancers. As observed from H&E staining (Figure 1a), BBN-induced bladder cancers were invasive. Furthermore, we found that XIAP expression was increased in BBN-induced mouse bladder cancer tissues compared with control tissues (Figure 1b), as evidenced by the statistical analysis (n = 10, *P* < 0.05) of XIAP relative expression level shown in Figure 1c. These data implied that XIAP expression might be related to BC invasion or metastasis.

To further evaluate whether the mice model findings extended to clinical samples, we performed IHC staining to examine XIAP expression in both non-metastatic bladder cancer (NMBC) and muscle-invasive bladder cancer (MIBC) tissues. As shown in Figure 1d, XIAP was weakly positive in non-invasive BC but strongly positive in invasive BC. Next, we sought to determine whether XIAP was correlated with tumor grades or stages, thus we analyzed the clinic-pathological characteristics of 96 bladder cancer patients (Table 1). The randomly chosen patient group was comprised of 43 patients with low-grade tumors and 53 with high-grade tumors. Staging criteria revealed that 92 of the 96 patients had NMBC while the remaining 4 had MIBC. XIAP was undetectable in normal urothelial cases and in 10 out of 43 low-grade BC cases. The majority of low-grade tumors (30/33, 90.9%) expressed low levels (1+) of XIAP, with only 3/33 (9.1%) expressing moderate levels (2+). In contrast, all high-grade tumors expressed XIAP, with 4 (7.5%) expressing low levels, 38 (71.7%) expressing moderate levels, and 11 (20.8%) expressing high levels (3+) (*P* < 0.0001). Statistically, the average of XIAP score in the high-grade group was 2.13 *vs*. 0.84 in the low-grade group (*P* < 0.0001) exhibiting a strong relationship between XIAP and high tumor grade. Based on staging, 1 out of 92 NMBC was XIAP-negative, with 37/92 (40.2%) expressing low XIAP levels, 38/92 (41.3%) expressing moderate levels and 7/92 (7.6%) expressing high levels. However, all 4 (100%) MIBC expressed high levels of XIAP. Overall, our findings suggest that XIAP expression might be related to bladder cancer invasion and metastasis.

**Table 1.** ﻿Clinicopathological characteristics of bladder cancer with XIAP expression.

			XIAP scores (n)				
		NO. of patients	0(n)	1+(n)	2+(n)	3+(n)	*P* Value
Tumor grade	Low	43	10	30	3	0	
	High	53	0	4	38	11	<0.0001
Stages	NMBC	92	10	37	38	7	
	MIBC	4	0	0	0	4	

### XIAP is upregulated through mRNA stability at its 3ʹUTR in invasive bladder cancer cells

**Figure 2.**
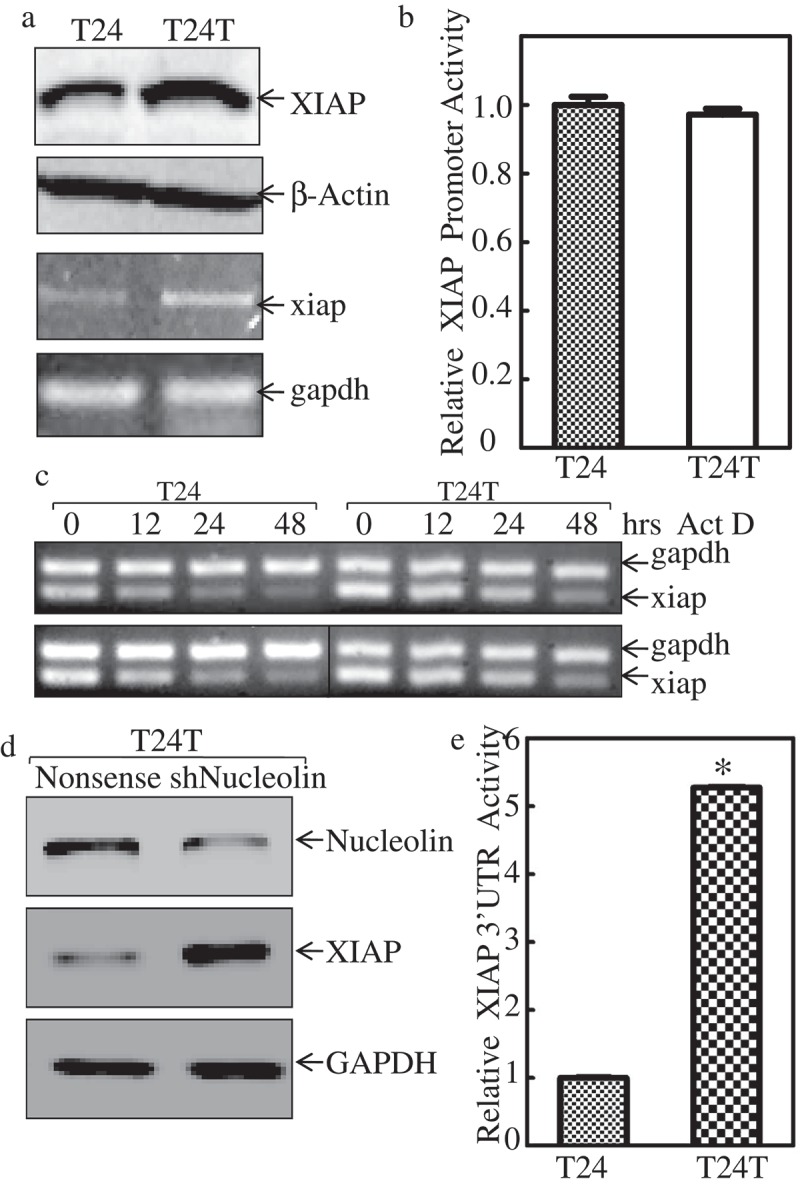
*XIAP* mRNA is stabilized in invasive bladder cancer cells. (a) Western Blot was applied to compare the protein expression levels of XIAP between the T24 and T24T (top two panels), β-Actin was used as internal control. RT-PCR was used to compare the mRNA level of *xiap* between these two cell lines (bottom two panels) and *gapdh* was used as internal control. The details of total cell protein extracts and mRNA obtained are described in ‘Material and Methods’. (b) Wild-type *xiap* promoter-driven luciferase reporter was co-transfected together with pRL-TK into T24 and T24T cells. Twenty-four hours post-transfection, the transfectants were extracted to evaluate luciferase activity. TK was used as internal control. The results were presented as *xiap* promoter activity relative to T24 cell, and each bar indicates mean±SD from three independent experiments. An asterisk (*) indicates a significant difference (*P* < 0.05). (c) *XIAP* mRNA stabilities were evaluated by RT-PCT in presence of Act D in T24 and T24T cells. (d) The cell extracts from T24T (nonsense) and T24 (shNucleolin) transfectants were subjected to Western blot as indicated, knockdown nucleolin can increase XIAP protein expression level. (e) xiap-3ʹUTR luciferase reporter was co-transfected together with pRL-TK into T24 and T24T cells, respectively. Twenty-four hours post-transfection, the transfectants were extracted to evaluate luciferase activity. TK was used as internal control. The results were presented as xiap-3ʹUTR activity relative to T24 cells, and each bar indicates mean±SD from three independent experiments. An asterisk (*) indicates a significant difference (*P* < 0.05).

Considering XIAP upregulation was related to BC invasion and metastasis. We aimed to understand the molecular mechanism underlying this upregulation and thus employed T24 cells and T24T cells with distinct metastatic characteristics for our *in vitro* studies. We compared XIAP expression in non-metastatic T24, and its metastatic derivative, T24T bladder cancer cell lines. As shown in Figure 2a, XIAP protein was highly expressed in T24T cells compared with T24 cells. To further test whether the increased protein level was due to mRNA upregulation, *xiap* mRNA level was compared in these paired cells. The results showed that xiap mRNA level was also higher in T24T cells than T24 cells, indicating that XIAP was regulated at the mRNA level, either *via* transcriptional level expression or mRNA stability level. To investigate the probability of transcriptional level expression, we transfected XIAP promoter-driven luciferase reporter and TK into these two cell lines and performed a luciferase assay to compare the promoter activity of XIAP between T24 and T24T cells. The data in Figure 2b showed that the two cells had similar XIAP promoter activity, which indicated that XIAP upregulation did not occur at the transcriptional expression level. To investigate the xiap mRNA stability level, we used Act D (Actinomycin D) to block the *de novo* mRNA synthesis to compare the half-life of xiap mRNA in these two cell lines. As shown in Figure 2c, we found that xiap mRNA was more stable in T24T than T24 cells. These data suggest that XIAP was upregulated *via* increased mRNA stability in invasive BC cells. AUF1, HuR, and nucleolin are known to affect mRNA stability. Our previous study found that only nucleolin was upregulated in T24T cells, while the other two proteins had minor changes [18]. To determine whether nucleolin was involved in upregulating xiap mRNA stability, we constructed stable cells line T24T in which nucleolin was knocked down (shNucleolin). Nucleolin knockdown efficiency is shown in Figure 2d. Interestingly, XIAP protein level was elevated when nucleolin was knocked down in T24T cells (Figure 2d), which was not consistent with our assumption that nucleolin stabilized xiap mRNA. Based on this unexpected result, we posited that noncoding RNAs might be involved in the regulation of xiap mRNA stability. It is well known that miRNAs can bind to mRNA 3ʹUTR region to affect mRNA stability [19,20]. To test the activity of xiap 3ʹUTR, we constructed XIAP 3ʹUTR luciferase reporter, and co-transfected with TK into T24 and T24T cells. The stable transfectants were used to compare the xiap 3ʹUTR activities between the two cells. The results showed that XIAP 3ʹUTR activity was much higher in T24T cells than T24 cells (Figure 2e). Together, these results indicated that xiap mRNA stability in metastatic T24T cells was upregulated through its 3ʹUTR.

### MiR-200c directly binds to XIAP 3ʹUTR to decrease its mRNA stability and inhibit bladder cancer cell invasion

**Figure 3.**
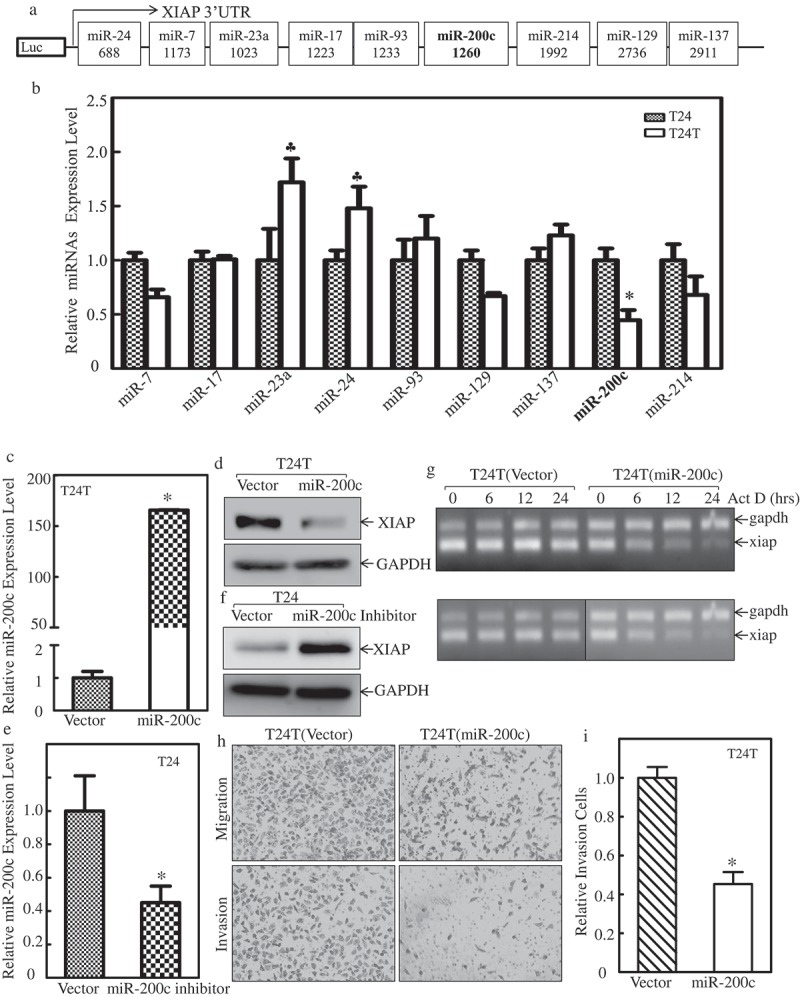
miR-200c binds to XIAP-3ʹUTR to decrease *XIAP* mRNA stability and inhibit cell invasion. (a) Potential miRNAs binding site in XIAP 3ʹ-UTR region. (b) Real-time PCR was applied to compare the expression level of these potential miRNAs in T24 and T24T cells. (c) Real-time PCR was used to identify the expression level of miR-200c in T24T (miR-200c) cells. (d) West blot (WB) was applied to detect the protein level of XIAP after miR-200c was overexpressed in T24T cells. (e) Real-time PCR was used to identify the expression level of miR-200c in T24 cells stably transfected with miR-200c inhibitor. (f) WB was applied to detect the protein level of XIAP after miR-200c inhibitor was transfected in T24 cells. (g) *XIAP* mRNA stabilities were evaluated by RT-PCT in presence of Act D in T24 and T24T cells. (h) T24T (vector) and T24T (miR-200c) cells were seeded to control or Matrigel inserts for trans-well invasion assay following the manufacturer’s instructions. The images were captured using an inverted microscope. (i) The invasion rate was normalized with the insert control according to the manufacturer’s instruction. An asterisk (*) indicates a significant difference between T24T (vector) and T24T (miR-200c) cells (*P* < 0.05). The values shown are mean ± SD from three independent experiments.

Based on the observation that xiap 3ʹUTR activity was upregulated in T24T cells, we used TargetScan to predict the potential miRNAs that can bind to 3ʹUTR of XIAP mRNA, and 9 putative miRNAs are shown in Figure 3a. We then used real-time PCR to determine the expression level of these 9 miRNAs (miR-7, miR-17, miR-23a, miR-24, miR-93, miR-129 miR-137, miR-200c, miR-214) in T24 and T24T cells. As shown in Figure 3b, miR-23a, miR-24 and miR-93 were elevated in T24T cells, miR-7 and miR-129 were slightly decreased in T24T cells, while miR-200c was dramatically decreased in T24T cells compared to T24 cells. Those results suggest that miR-200c might have a role in regulating XIAP mRNA stability in T24T cells.

To confirm the hypothesis that miR-200c might be responsible for XIAP mRNA stability, we constructed stable transfectants of miR-200c overexpression in T24T cells and miR-200c inhibition in T24 cells. The stabilized cell lines were identified as shown in Figure. 3c and 3e. After miR-200c was overexpressed in T24T cells, XIAP protein was decreased compared with its vector control (Figure 3d); conversely, when we inhibited miR-200c in T24 cells, XIAP was upregulated (Figure 3f). To determine whether miR-200c inhibited *xiap* at mRNA stability level, T24T (vector) and T24T (miR-200c) cells were treated with the *de novo* mRNA synthesis inhibitor Act D, and the decay rate of *xiap* mRNA was assessed by RT-PCR (Top panel of Figure 3g). To make basal levels of mRNA comparable between T24T (vector) and T24T (miR-200c) cells, we adjusted intensity of RT-PCR products in the T24T (miR-200c) cells (as seen in *gapdh* levels of bottom panel). As shown in Figure 3g, *xiap* mRNA stability was significantly reduced in T24T (miR-200c) cells. Our results showed that miR-200c overexpression attenuated *xiap* mRNA stability in BCs. To investigate whether miR-200c had an impact on cancer cell invasion, a trans-well assay was performed using T24T (vector) and T24T (miR-200c) cells. The results showed that overexpression of miR-200c significantly inhibited bladder cancer cell invasion (Figure 3h and 3i). Altogether, this data indicates that decreased miR-200c resulted in *xiap* mRNA stabilization and increased BC invasion.

### P-CREB binds to miR-200c promoter and contributes its transcription

**Figure 4.**
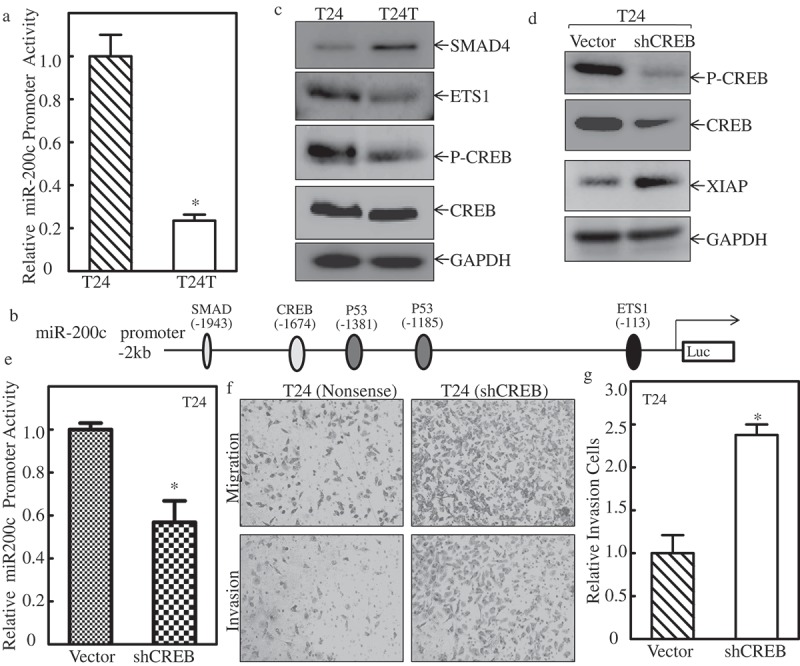
p-CREBcontributes tomiR-200ctranscription and inhibits XIAP expression and cancer cell invasion of bladder cancer cells. (a) T24 and T24T cells were co-transfected withmiR-200c promoter-driven luciferase reporter and pRL-TK. Twenty-four hours after transfection, the cells were extracted and luciferase activity was determined. The results are presented as miR-200c promoter activity relative to T24 cells with normalized by internal TK activity. An asterisk (*) indicates a significant difference between the paired cells (*P* < 0.05). The bars are mean ± SD from three independent experiments. (b) Potential transcription factors binding site in miR-200c promoter region. (c) WB applied to detect the transcription factors, CREB, SMAD4, and ETS1 expression between T24 and T24T cells. GAPDH was used as internal control. (d) XIAP expression level was evaluated using Western Blot after knockdown of CREB in T24 cells. (e) miR-200c expression level was evaluated using real time-PCR after knockdown of CREB in T24 cells. (f) T24 (nonsense) and T24 (shCREB) cells were seeded to control or Matrigel inserts for transwell invasion assay following the manufacturer’s instructions. The images were captured using an inverted microscope. (g) The invasion rate was normalized with the insert control according to the manufacturer’s instruction. An asterisk (*) indicates a significant difference between T24/Nonsense and T24/shCREB cells (*p* < 0.05). The values shown are mean ± SD from three independent experiments.

Given our results showing that miR-200c downregulation was important for XIAP upregulation in BC, our subsequent efforts were spent on understanding the mechanisms underlying miR-200c downregulation in metastatic T24T cells. We first examined the transcription level of miR-200c between these two cells. The miR-200c promoter-driven luciferase reporter (about 2 kb, was kindly gifted by Dr. Hung) [21] and TK were co-transfected into T24 and T24T cells, and the miR-200c promoter activity was determined using luciferase assay. The results in Figure 4a showed that the miR-200c promoter activity was dramatically decreased in T24T cells compared with T24 cells, suggesting that miR-200c was regulated at the transcriptional level. Therefore, the potential transcription factors that can bind to the miR-200c promoter region, including p-CREB, CREB, SMAD4, and ETS1 (Figure 4b) were analyzed, and results are shown in Figure 4c. Since p53 is mutated in bladder cancer T24/T24T cells, we did not show its expression here. The results demonstrated that SMAD4 expression was elevated in T24T cells, and was not consistent with miR-200c downregulation in T24T cells. ETS1 was slightly reduced, and p-CREB was significantly decreased in T24T cells, with no impact on total CREB between T24 and T24T cells. These results suggested that p-CREB might be the transcription factor target of miR-200c transcription.

To further define the role of p-CREB in miR-200c expression in T24T cells, CREB-specific shRNA was used to knockdown CREB expression in T24 cells, and the knockdown efficiency is shown in Figure 4d. The protein level of XIAP expression was increased remarkably when CREB was knocked down in T24 cells (Figure 4d). Consistently, the miR-200c promoter activity was depressed in T24 (shCREB) cells (Figure 4e). Importantly, the cell invasion activity of T24 (shCREB) cells were increased profoundly compared with T24 (nonsense) cells (Figure. 4f & 4g). These results suggest that downregulation of transcriptional factor CREB resulted in miR-200c reduction and XIAP overexpression, which in turn promoted cell invasion in bladder cancer cells.

### XIAP expression is critical for T24T cell lung metastasis in vivo

**Figure 5.**
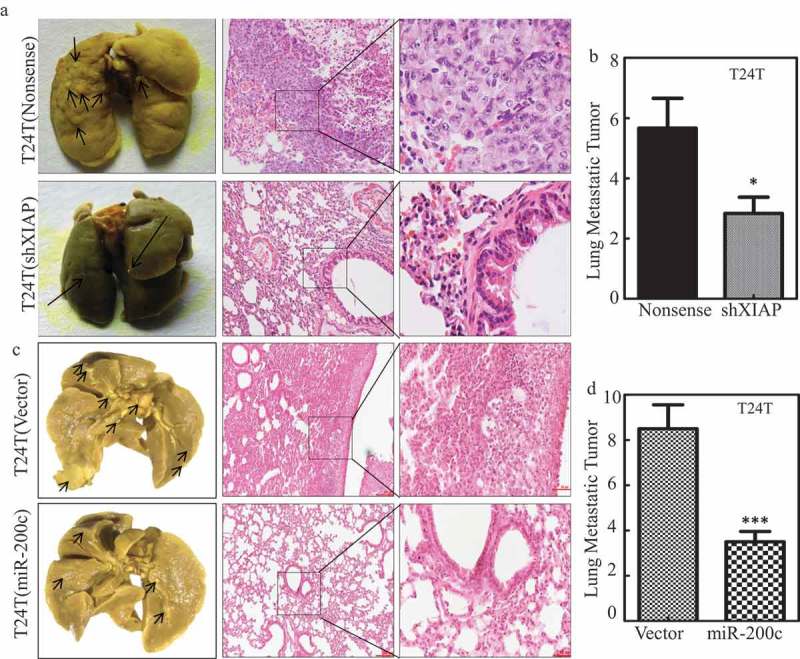
MiR-200c and XIAP are critical for T24T cell lung metastasis. (a and c) Representative images of lungs and lung surface metastatic foci as indicated are shown after fixation in a neutral-buffered formalin/Bouin’s fixative solution; histologic appearance of lung metastases: H&E-stained sections (H&E, ×40 and ×100). (b and d) The lung metastatic tumor number in the group mice that were injected with T24T (nonsense) *vs*. T24T (shXIAP), T24T (vector) *vs*. T24T (miR-200c), respectively.

To evaluate the contribution of XIAP overexpression in mediating BC lung metastasis, T24T (nonsense) and T24T (shXIAP) stable transfectants were injected into nude mice *via* the lateral tail vein and the lung metastatic abilities of two transfectants were evaluated. The results showed that knockdown of XIAP in T24T cells increased the mouse survival rate in comparison to T24T (nonsense) cells (Table 2). Consistent with mouse survival rate, the number and size of lung metastatic tumors in mice injected with T24T (shXIAP) cells were decreased remarkably (Table 2 and Figure 5a and 5B). Moreover, overexpression of miR-200c in T24T cells, T24T (miR-200c), attenuated the *in vivo* lung metastatic ability with prolonged mouse survival (Table 3 and Figure 5c and 5d), revealing that miR-200c was able to inhibit T24T cell lung metastasis. These results strongly demonstrate that miR-200c downregulation can act as a positive upstream regulator responsible for promoting XIAP expression, and BC lung metastasis.

**Table 2.** Role of XIAP in BC lung metastasis in nude mice.

Cell Type	T24T (nonsense)	T24T(shXIAP)
Mouse death by 180 days	3/6 (50%)	1/6 (14.2%)
Lung metastatic tumor number/mouse	5.7 ± 1.0	2.8 ± 0.5
Lung metastatic tumor size	Relative big	Small

**Table 3.** Role of miR-200c in BC lung metastasis in nude mice.

Cell Type	T24T(vector)	T24T(miR-200c)
Mouse death by 180 days	4/8 (50%)	1/8 (12.5%)
ung metastatic tumor number/mouse	8.5 ± 1.1	3.5 ± 0.5
Lung metastatic tumor size	Relative big	Small

## Conclusions

Muscular invasive bladder cancer represents a major therapeutic challenge of this disease; therefore, understanding the mechanisms underlying invasion and progression are crucial to potential drug discovery against the invasive malignant disease. In the current studies, we explored XIAP could promote invasion and lung metastasis of human BCs through RhoGDIb/MMP-2 pathway [10,11], but the molecular mechanism of XIAP upregulation in human BC remains unclear. Our result showed that XIAP was overexpressed in human high-grade BCs, high metastatic human T24T cells, and in mouse invasive BCs. Mechanistic studies indicated that XIAP overexpression in high metastatic T24T cells was due to the increased XIAP mRNA stability, which was mediated by the reduction of the decreased miR-200c binding to 3ʹUTR region of XIAP mRNA, whereas CREB inactivation was responsible for attenuated miR-200c transcription in human BC cells. Such miR-200c-mediated overexpressed XIAP promoted invasion *in vitro* and lung metastasis *in vivo* of T24T cells. Our results demonstrate the molecular basis leading to XIAP overexpression and its crucial role in BC invasion *in vitro* and lung metastasis in nude mice.

XIAP is a member of the IAP family, and has three repeats of the baculovirus IAP repeat (BIR) domain at its NH2 terminus and a RING finger domain near its COOH terminus [22]. It has been reported that XIAP is overexpressed in acute and chronic leukemia [23,24], prostate cancer [25], breast cancer [26–28], and many other cancers [29–31]. In addition to numerous studies elucidating the mechanisms of XIAP’s anti-apoptotic function, our recent studies have revealed several non-apoptosis-related functions of XIAP and its RING domain, such as upregulation of Cyclin D1 to promote bladder cancer cell growth [6] and promotion of colon cancer cell invasion *via* inhibition of RhoGDIα SUMOylation at lys-138 [7]. XIAP’s function as a metastatic driver by its activation of the NFκB pathway *via *E3 Ligase activity in human prostate cancer cells [8]. Another study report that XIAP-mediated ubiquitination regulates C-RAF kinase, which, as the effector protein of Ras, initiates MAPK cascades and thereby mediates cell growth and migration [32]. Nevertheless, the overall role of XIAP in cancer progression might be dependent on cancer tissues and cell types. Our present study revealed that XIAP was elevated in BBN-induced mouse BC tissues (Figure 1b) and human bladder cancer tissues (Figure 1d). We also found that XIAP expression was positively related to the tumor grades and muscle-invasive tumors from XIAP staining in human BCs (Table 1). To further study the relationship between XIAP and bladder cancer metastasis, we knocked down XIAP in T24T cells and utilized the T24T shXIAP and its nonsense control cells in an *in vivo* lung metastasis assay. Those results showed that T24T (shXIAP) cells exhibited dramatic inhibition of lung metastasis ability compared with T24T nonsense cells. Based on these observations, we conclude that XIAP plays an oncogenic role to promote BC invasion and metastasis.

For the mechanism study, we aimed to understand how XIAP regulated in metastatic BCs. We found that XIAP was upregulated at mRNA stability, and the 3ʹUTR region was responsible for the mRNA stabilization. By screening the potential miRNAs, miR-200c was defined as a direct interacting with the 3ʹUTR region of XIAP mRNA, which is supported from previous studies indicating the direct binding miR-200c to its binding site in the 3ʹUTR region of XIAP mRNA [14]. In addition, both overexpression of miR-200c in T24T cells and inhibition of miR-200c in T24 cells confirmed that miR-200c could downregulate XIAP expression, suggesting that miR-200c is directly upstream regulator responsible for XIAP overexpression in human BC cells.

MiR-200c is a member of the miR-200 family, which contains miR-200a, miR-200b, miR-141, miR-429, and all these miRNAs are involved in regulating cancer metastasis. MiR-200c also acts as a considerable modulator in the process of EMT [33,34], a cell development regulating process that affects tumor development and metastasis [13,14]. It has been reported that miR-200c suppresses many cancer metastasis properties, for example, by inhibiting MMP-3 expression to attenuate ovarian cancer metastasis [15], downregulating E-cadherin to affect EMT in human renal cell carcinoma [16], and targeting the 3ʹUTR region of JNK2 to reduce the metastasis ability of colorectal cancer [35]. Moreover, in bladder cancer, it has been reported that miR-200c is downregulated in cancer specimens compared with adjacent tissues in the same patients [14], and miR-200c could inhibit bladder cancer cell invasion through direct targeting of BMI-1 and E2F3. In the current study, we found that miR-200c could directly target the 3ʹUTR region of XIAP to decrease the invasive capability of bladder cancer. In addition, overexpression of miR-200c could inhibit T24T cells lung metastasis in nude mice. These results strongly indicate that miR-200c is a tumor suppressor miRNA that can restrain bladder cancer metastasis through degradation of *xiap* mRNA.

We further elucidate the upstream regulator that mediates miR-200c the transcriptional modulation in human BC cells. After screening potential transcription factors, we found that p-CREB was responsible for miR-200c transcription in highly invasive bladder cancer cells. CREB (cAMP response element-binding protein) is a transcription factor that binds to its targeted gene, thereby increasing or decreasing transcription of its targeted genes [36]. CREB protein has been well studied, and has many functions in several organs [37,38].In a previous study, we found that p27 suppresses COX-2 expression by inhibiting CREB phosphorylation upon arsenate exposure [39]. To the best of our knowledge, there is no report about the relationship between CREB and bladder cancer invasion. In our current study, we found that p-CREB was the transcription factor of miR-200c and was downregulated in invasive bladder cancer cells (Figure 4c). Knockdown of CREB in T24 cells could increase its invasion ability accompanied with reduction of miR-200c transcription (Figure 4d-4g). From these data, we conclude that CREB acts as a positive upstream regulator and binds to the promoter region of miR-200c to increase its transcription and subsequently inhibits XIAP expression and bladder cancer invasion. Thus, downregulated CREB results in miR-200c downregulation and in turn promoting XIAP overexpression and BC invasion and lung metastasis in human BC cells. In future studies, we aim to focus on the mechanisms underlying p-CREB decrease in invasive bladder cancers.

Collectively, our findings demonstrate the molecular basis leading to XIAP overexpression and the crucial role of XIAP in BC invasion *in vitro* and lung metastasis in nude mice.

## Material and methods

### Cell lines, plasmids, and antibodies

T24 and T24T cells were described in our previous studies [18,40], and they were cultured in DMEM: F-12 = 1:1 with 10% FBS (ATLANTA, Flowery Branch, GA, USA). The XIAP promoter-driven luciferase reporter was constructed into PGL3 Basic vector using primers, Forward: 5ʹ-CGG GGT ACC TAC TTA TTG CCA CTG AAA AA-3ʹ, Reverse: 5ʹ-CCG CTC GAG TGG CCC CAG CCT AGG TGA AG-3ʹ. The XIAP 3ʹUTR luciferase reporter was constructed into Pmir-report by using primers, Forward:5ʹ-CCG CTC GAG AAG GCT TAG GCA TGT TCA AAC GC-3ʹ, Reverse:5ʹ-CTA GAC TAG TAG GTT TGT TGT GAG AAT CTT GTA-3ʹ. The miR-200c promoter-driven luciferase reporter was kindly gifted by Dr. Hung (The University of Texas MD Anderson Cancer Center, Houston, TX, USA). The constructs of short hairpin RNA specific to XIAP (shXIAP), nucleolin (shNucleolin), and their nonsense control constructs were purchased from Open Biosystem (Pittsburg, PA, USA). The plasmid expression miR-200c was purchased from Addgene. The hsa-miR-200c inhibitor plasmid was purchased from GeneCopoeia (Rockville, MD, USA). Plasmids were prepared using the Plasmid Preparation/Extraction Maxi kit from QIAGEN (Valencia, CA, USA). The specific antibodies for β-Actin (sc-70,319) and Smad4 (sc-7966) were purchased from Santa Cruz Biotechnology, Inc. (Santa Cruz, CA, USA). CREB (9197S), phosphor-CREB (9198S), and ETS1 (14069S) antibodies were purchased from Cell Signaling Technology (Beverly, MA, USA). XIAP (610,763) antibody was purchased from BD Biosciences (San Jose, CA, USA). GAPDH (GTX100118) antibody was purchased from Genetex (Irvine, CA, USA). Antibodies against Nucleolin (N2662) were obtained from Sigma (St. Louis, MO, USA). Actinomycin D (Act D) was purchased from Santa Cruz (Dallas, TX, USA).

### Cell transfection

Cell transfections were conducted using PolyJet^TM^ DNA *in vitro* Transfection Reagent (SignaGen Laboratories, Rockville, MD, USA) according to the manufacturer’s instructions. For the stable cell line selection, cells were subjected to Hygromycin B (200–400 μg/mL), G418 (500–1000 μg/mL), or Puromycin (0.2–0.3 μg/mL) treatment depending on the different antibiotic resistance plasmids transfected. Subsequently, cells surviving from the antibiotics selection were pooled as stable mass transfectants.

### Western blot analysis

Whole cell extracts were prepared using the cell lysis buffer (10 mM Tris-HCl, pH 7.4, 1% SDS, and 1 mM Na_3_VO_4_) as described in our previous studies [41]. Proteins were resolved by SDS-PAGE, transferred to membranes, blocked with 5% dry milk, then probed with the indicated primary antibodies, and incubated with the AP-conjugated secondary antibody. Signals were detected by the enhanced chemifluorescence Western blotting system as described in previous reports [35,40,42]. The images were acquired by scanning with the Phosphoimager Typhoon FLA 7000 (GE, Pittsburgh, PA, USA)

### Luciferase promoter reporter assay

XIAP promoter luciferase reporter, XIAP 3ʹUTR luciferase reporter or miR-200c promoter luciferase reporter and pRL-TK were each transiently co-transfected into T24 and T24Tcells. Twenty-four hours later, luciferase activity was determined using the Luciferase Assay System kit (Promega, Madison, WI, USA) as described in our previous studies [35,42,43]. The values were normalized by internal TK signal. All experiments were conducted in triplicate and the results expressed as mean ± standard error.

### RT-PCR

Total RNA was extracted using the TRIzol reagent (Invitrogen, Grand Island, NY, USA) as described in the manufacturer’s instructions. Next, 5 μg total RNA was used for first-strand cDNA synthesis with oligdT primer by SuperScript^TM^ First-Strand Synthesis system (Invitrogen, Grand Island, NY, USA) as described in our previous studies [43,44]. Specific primers (Invitrogen, Grand Island, NY, USA) were used for PCR amplification. The primers used in this study were: *human xiap* (Forward: 5ʹ-cgg ctg tcc tgg cgc gaa aa-3ʹ, Reverse: 5ʹ-acc ctg ctc gtg cca gtg ttg-3ʹ), and *human gapdh* (Forward: 5ʹ-gat gat ctt gag gct gtt gtc-3ʹ, Reverse: 5ʹ-cag ggc tgc ttt taa ctc tg-3ʹ).

### Quantitative RT-PCR for miRNA assay

The total cell microRNAs and human bladder tissue microRNAs were extracted using the miRNeasy Mini Kit (Qiagen, Valencia, CA, USA) as described in our previous studies [45,46]. Total RNA (1 μg) was used for reverse transcription, and analysis of miR-7, miR-17, miR-23a, miR-24, miR-93, miR-129, miR-137, miR-200c, and miR-214 expression were determined by the 7900HT Fast Real-time PCR system (Applied Biosystems, Carlsbad, CA, USA) using the miScript PCR kit (Qiagen, Valencia, CA, USA). The primers for microRNA were purchased from Invitrogen (Carlsbad, CA, USA), and *U6* was used as a control. Cycle threshold (CT) values were determined, and the relative expression of microRNAs was calculated using the values of 2^−ΔΔCT^.

### Cell migration and invasion assay

The control inserts without Matrigel and invasion kit were purchased from BD Falcon; the invasion assay was performed according to the manufacturer’s instruction in normal cell culture serum as described in our previous studies [18,45]. The cells were seeded in the inserts both with and without Matrigel. After incubation in transwell, the cells in the apical and basal chambers were fixed with 3.7% formalin, 100% methanol, Giemsa (1:20 diluted with PBS). Cells were washed twice with PBS, the non-invaded cells remaining on the apical chamber were then scraped off with a cotton swab (PBS wetted). The cells migrating through control insert membranes were counted as migration ability. The invasion ability was measured as the ratio of the cells invading through Matrigel insert membrane to the cells migrating through control insert membrane. Images were acquired with an Olympus DP71, and the number of the cells was calculated by Image J software (NIH, Bethesda, MD, USA).

### Animal experiments and bladder tissue

C57BL/6 mice at age 6 to 8 weeks were randomly divided into two groups as indicated: normal control, and treated with 0.5% BBN in water. After 6 months, the mice were then euthanized, and mouse bladders were removed to evaluate bladder pathology and IHC staining.

### IHC paraffin embedding of mice and human bladder specimens

Tumor tissues obtained from the mice and clinical patients were fixed in formalin and paraffin-embedded. For the IHC assays, antibodies specific against XIAP were obtained from BD (San Jose, CA, USA) or K5 (Cell Signaling Technology). The resultant immunostaining images were captured using the AxioVision Rel.4.6 computerized image analysis system (Carl Zeiss). Protein expression levels were analyzed by calculating the integrated optical density (IOD) per stained area using Image-Pro Plus version 6.0 (Media Cybernetics).

### Human urothelial carcinoma tissue array

Tissue Samples and Tissue Microarray Construction: After Institutional Review Board approval (protocol# WMC, L-10,884), a total 60 cases with a diagnosis of urothelial carcinoma were obtained from files of the Pathology Department of Westchester Medical Center. In all cases, tissues were fixed in neutral buffered formalin and embedded in a paraffin block as routine surgical pathology procedure. For tissue microarray construction, three cores (1.0 mm in diameter) of each tumor from patient paraffin blocks were taken from donor paraffin blocks. The final tissue microarrays consisted of up to 180 tissue cores per slide with a 1.5 mm space between each core. After construction, 4 μm sections were cut and H&E staining was performed on the initial slide to verify the histology.

IHC: After initial deparaffinization, endogenous peroxidase activity was blocked with 0.3% H_2_O_2_. Deparaffinized sections were microwaved in 10 mMol/L citrate buffer (pH 6.0). The slides were then incubated for 1 h at room temperature using goat polyclonal-specific antibodies against XIAP (1:100; BD 610,763) followed by biotinylated rabbit anti-goat IgG (Vector Laboratories, Burlingame, CA, USA) for 30 min, and finally AB Complex (Vector Laboratories, Burlingame, CA, USA). The antibody-bound complex was visualized with 0.125% amino-ethyl carbazole (AEC, Sigma, St Louis, MO, USA) and 0.003% (v/v) H_2_O_2_. The sections were then counterstained in Mayer’s hematoxylin. For negative controls, the primary antibody was replaced with normal IgG.

### T24T cell lung metastatic assay

All animal studies were performed in the animal institute of Wenzhou Medical University according to the protocols approved by the Medical Experimental Animal Care Commission of Wenzhou Medical University. Female euthymic (nu+/nu+) mice were purchased from Shanghai Silaike Experimental Animal Company, Ltd. (license No.SCXK, Shanghai 2010 0002; Shanghai, China). A total of 24 mice at age 5–6 weeks were randomly divided into each group, and transfectants of T24T (nonsense) vs T24T (shXIAP), and T24T (vector) vs T24T (miR-200c) were injected into nude mice *via* the lateral tail vein (2 × 10^6^ cells in 100μL PBS/mouse). The mice were evaluated and weighed twice a week. The lungs were removed by dissection after natural death or euthanization at indicated times after injection. Six lungs from the indicated groups were fixed in Bouin’s fixative solution (Sigma Aldrich) for 24 h and the numbers of lung surface metastatic lesions were counted on each lobe of every specimen. The left lungs in each group were fixed and embedded in paraffin for histopathological evaluation.

### Statistical analysis

Student’s *t* test was utilized to determine significant differences. The differences were considered to be significant at a *P* ≤ 0.05.
